# Supplementary material for: Causal interplay between lactose intolerance and gut microbiota: a combined bidirectional Mendelian randomization and in vivo validation study
Source: Front Nutr. 2026 Jun 1;13:1803337. doi: 10.3389/fnut.2026.1803337 (PMC13265576; doi:10.3389/fnut.2026.1803337)
Supplement: Supplementary file 1 [file Data_Sheet_1.zip › supplementary materials/Forward/leave one out cross validation/ebi-a-GCST90027754.finngen_R12_E4_LACTONAS.pdf]

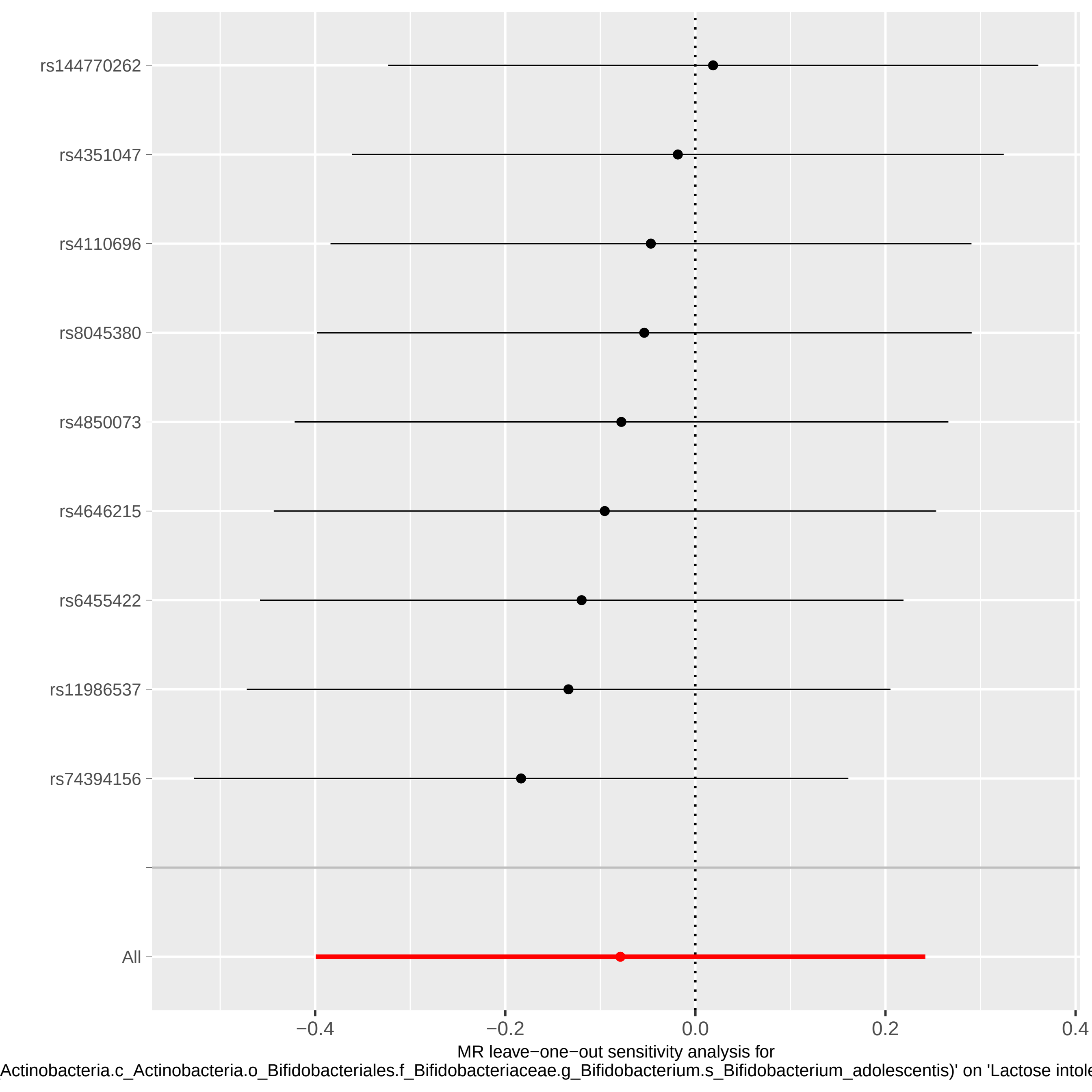
rs144770262
rs4351047
rs4110696
rs8045380
rs4850073
rs4646215
rs6455422
rs11986537
rs74394156
All
−0.4
−0.2
0.0
0.2
0.4
MR leave−one−out sensitivity analysis for
Actinobacteria.c_Actinobacteria.o_Bifidobacteriales.f_Bifidobacteriaceae.g_Bifidobacterium.s_Bifidobacterium_adolescentis)' on 'Lactose intole
